# Digitalis to Prevent Excessive Desire for Copulation

**Published:** 1884-09

**Authors:** 


DIGITALIS TO PREVENT EXCESSIVE DESIRE FOR COPULATION.

In the case of a man who had persistent desire to have intercourse with his wife, even during the day, Dr. Folsom reports in the *Med. World* the successful action of digitalis as a complete depressor of venereal appetite. He prescribed it in ten drop doses, but the efficient point was attained only after a teaspoonful at a dose was reached, which abolished the power to cause erections, though it did not prevent the amative desire. In another case a young man wanted something that would enable him to hold his passion in check. The digitalis was prescribed for him also, commencing with ten drops, gradually increasing the dose until the desired effect was obtained, which required teaspoonful doses, as in the previous case. Dr. Folsom prescribes it in insanity resulting from self-abuse.

